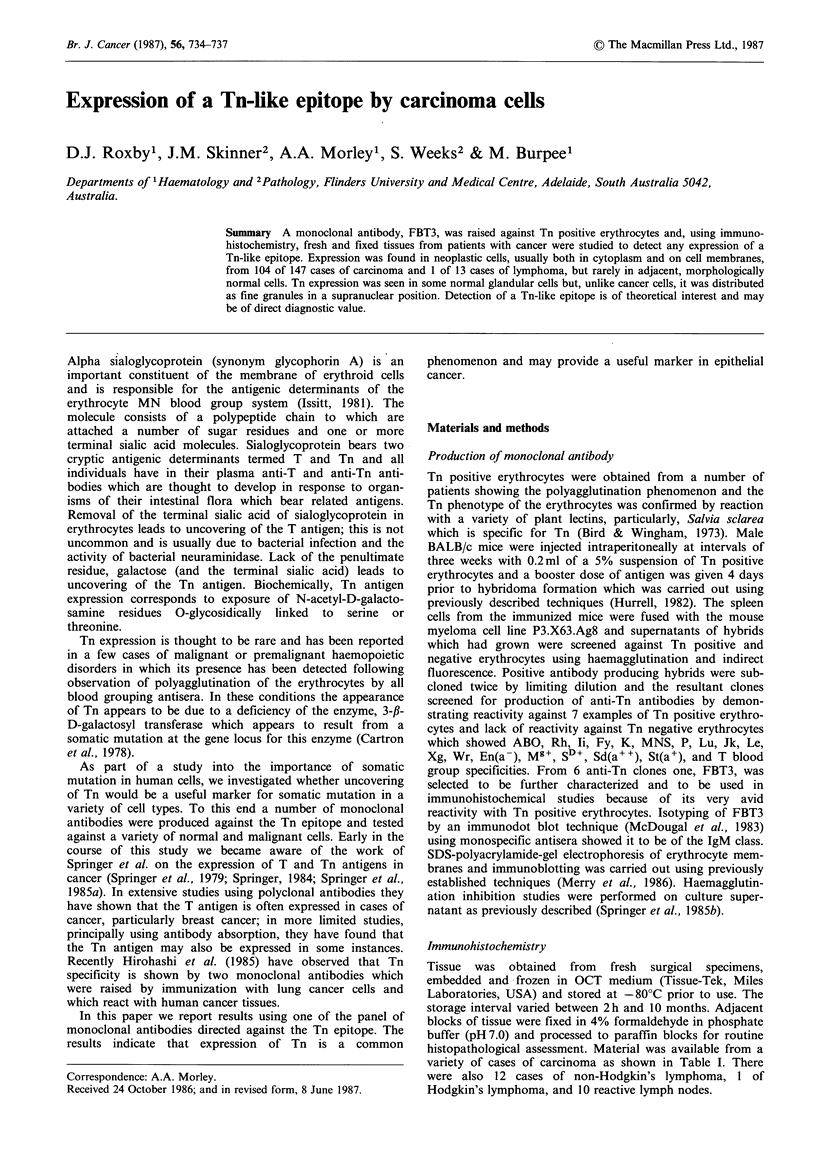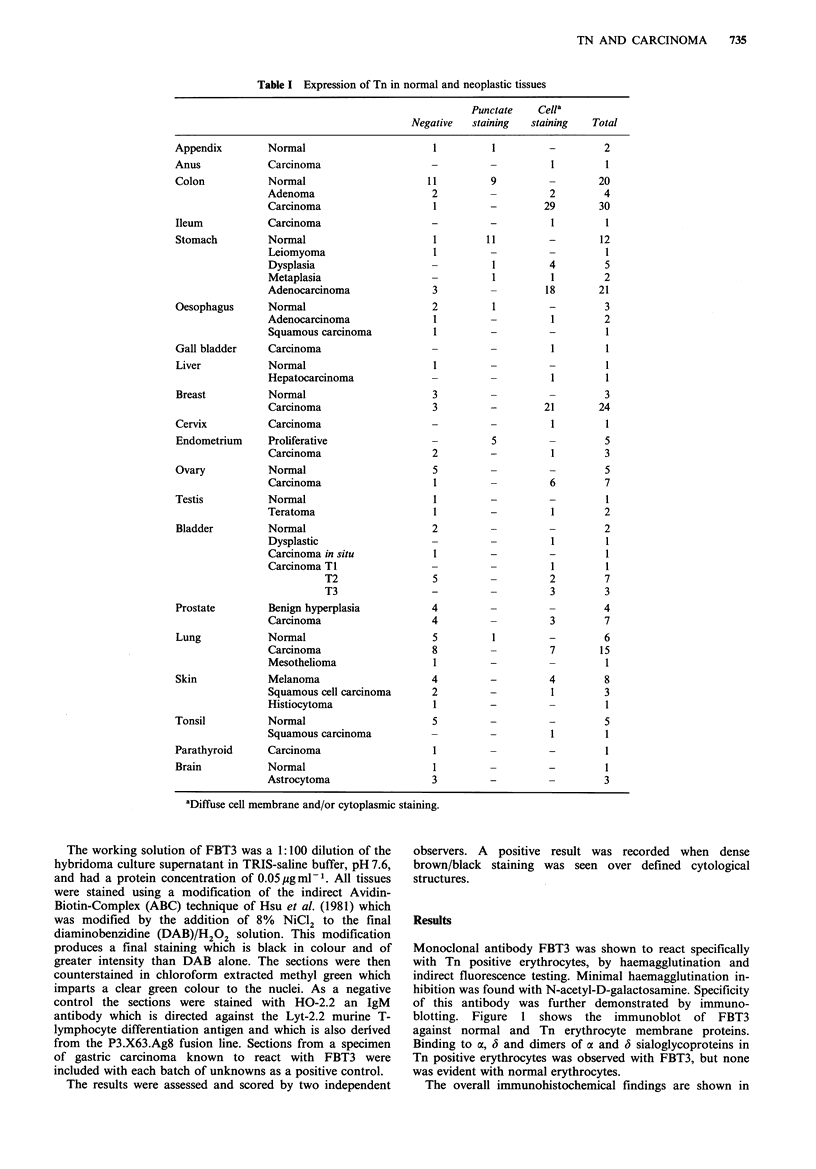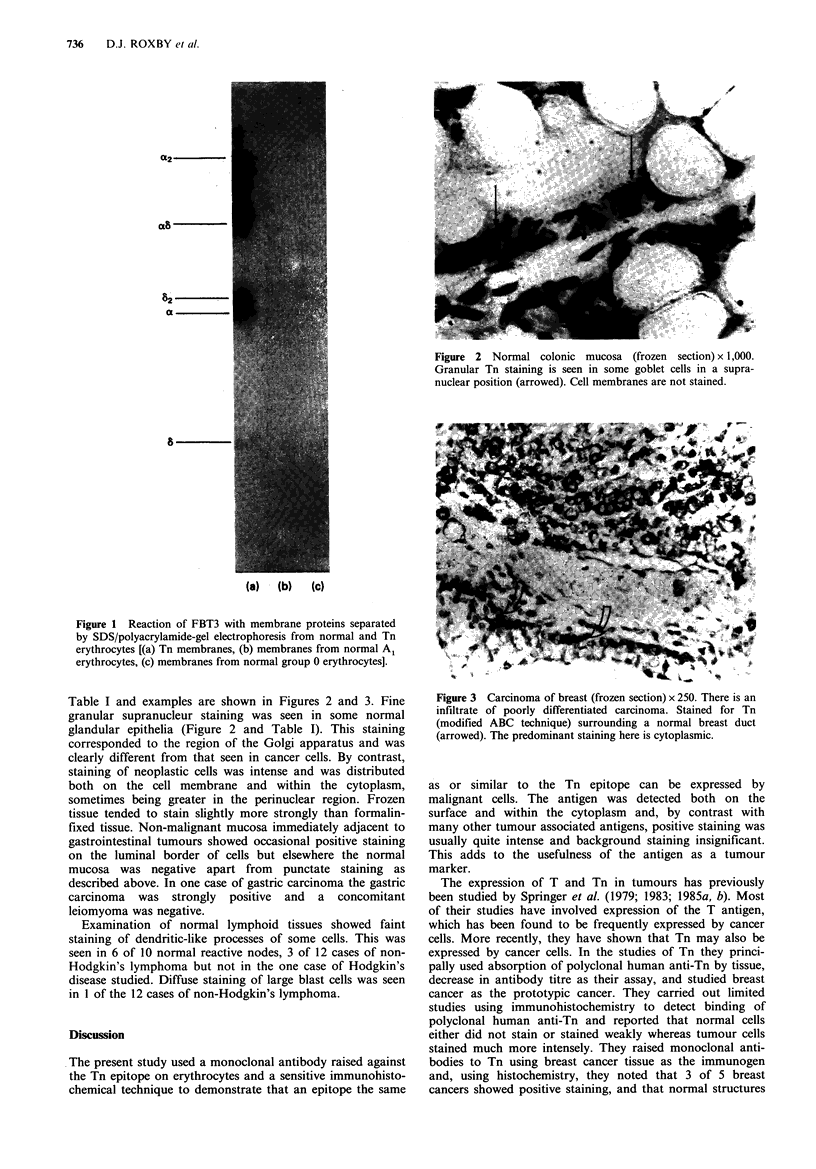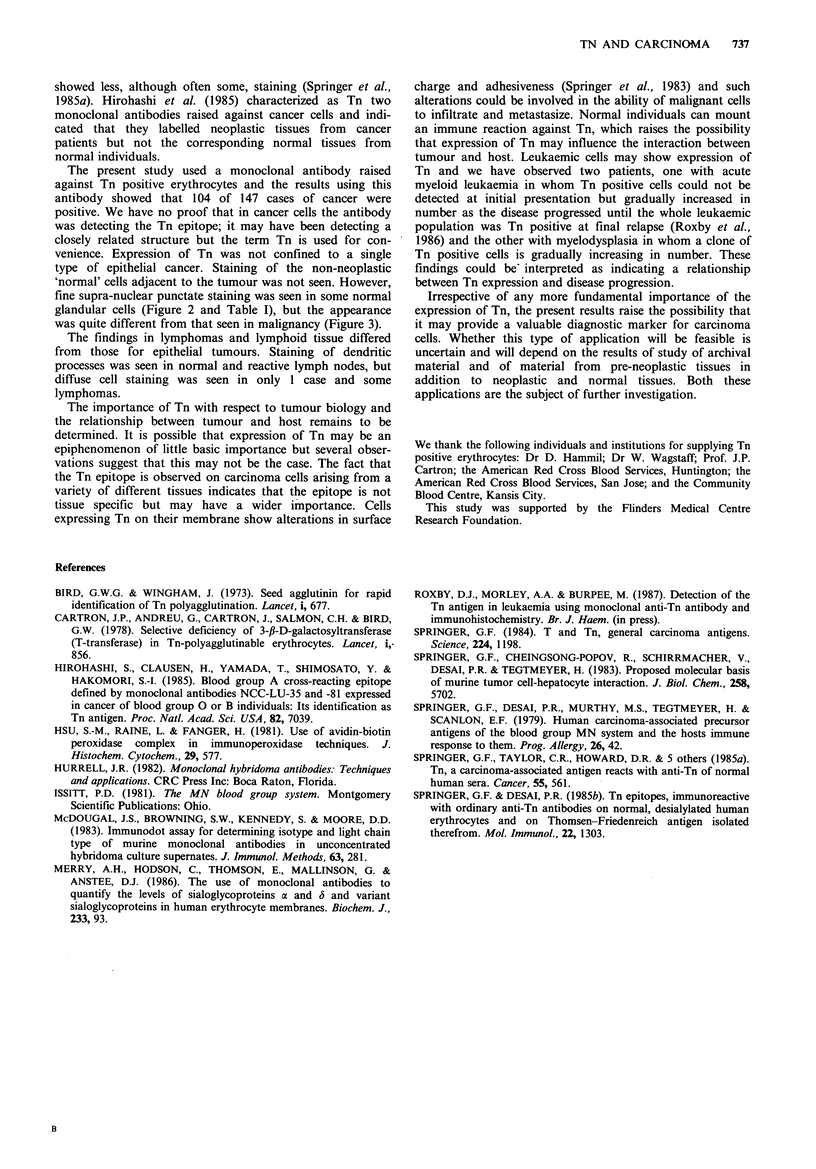# Expression of a Tn-like epitope by carcinoma cells.

**DOI:** 10.1038/bjc.1987.279

**Published:** 1987-12

**Authors:** D. J. Roxby, J. M. Skinner, A. A. Morley, S. Weeks, M. Burpee

**Affiliations:** Department of Haematology, Flinders University, Adelaide, South Australia.

## Abstract

**Images:**


*Br. J. Cancer* (1987), **56**, 734–737 © The Macmillan Press Ltd., 1987

### Expression of a Tn-like epitope by carcinoma cells

D.J. Roxby[1], J.M. Skinner[2], A.A. Morley[1], S. Weeks[2] & M. Burpee[1]

*Departments of [1]Haematology and [2]Pathology, Flinders University and Medical Centre, Adelaide, South Australia 5042, Australia.*

**Summary** A monoclonal antibody, FBT3, was raised against Tn positive erythrocytes and, using immunohistochemistry, fresh and fixed tissues from patients with cancer were studied to detect any expression of a Tn-like epitope. Expression was found in neoplastic cells, usually both in cytoplasm and on cell membranes, from 104 of 147 cases of carcinoma and 1 of 13 cases of lymphoma, but rarely in adjacent, morphologically normal cells. Tn expression was seen in some normal glandular cells but, unlike cancer cells, it was distributed as fine granules in a supranuclear position. Detection of a Tn-like epitope is of theoretical interest and may be of direct diagnostic value.

Alpha sialoglycoprotein (synonym glycophorin A) is an important constituent of the membrane of erythroid cells and is responsible for the antigenic determinants of the erythrocyte MN blood group system (Issitt, 1981). The molecule consists of a polypeptide chain to which are attached a number of sugar residues and one or more terminal sialic acid molecules. Sialoglycoprotein bears two cryptic antigenic determinants termed T and Tn and all individuals have in their plasma anti-T and anti-Tn antibodies which are thought to develop in response to organisms of their intestinal flora which bear related antigens. Removal of the terminal sialic acid of sialoglycoprotein in erythrocytes leads to uncovering of the T antigen; this is not uncommon and is usually due to bacterial infection and the activity of bacterial neuraminidase. Lack of the penultimate residue, galactose (and the terminal sialic acid) leads to uncovering of the Tn antigen. Biochemically, Tn antigen expression corresponds to exposure of N-acetyl-D-galactosamine residues O-glycosidically linked to serine or threonine.

Tn expression is thought to be rare and has been reported in a few cases of malignant or premalignant haemopoietic disorders in which its presence has been detected following observation of polyagglutination of the erythrocytes by all blood grouping antisera. In these conditions the appearance of Tn appears to be due to a deficiency of the enzyme, 3-$\beta$-D-galactosyl transferase which appears to result from a somatic mutation at the gene locus for this enzyme (Cartron *et al.*, 1978).

As part of a study into the importance of somatic mutation in human cells, we investigated whether uncovering of Tn would be a useful marker for somatic mutation in a variety of cell types. To this end a number of monoclonal antibodies were produced against the Tn epitope and tested against a variety of normal and malignant cells. Early in the course of this study we became aware of the work of Springer *et al.* on the expression of T and Tn antigens in cancer (Springer *et al.*, 1979; Springer, 1984; Springer *et al.*, 1985*a*). In extensive studies using polyclonal antibodies they have shown that the T antigen is often expressed in cases of cancer, particularly breast cancer; in more limited studies, principally using antibody absorption, they have found that the Tn antigen may also be expressed in some instances. Recently Hirohashi *et al.* (1985) have observed that Tn specificity is shown by two monoclonal antibodies which were raised by immunization with lung cancer cells and which react with human cancer tissues.

In this paper we report results using one of the panel of monoclonal antibodies directed against the Tn epitope. The results indicate that expression of Tn is a common phenomenon and may provide a useful marker in epithelial cancer.

Correspondence: A.A. Morley.
Received 24 October 1986; and in revised form, 8 June 1987.

### Materials and methods

### *Production of monoclonal antibody*

Tn positive erythrocytes were obtained from a number of patients showing the polyagglutination phenomenon and the Tn phenotype of the erythrocytes was confirmed by reaction with a variety of plant lectins, particularly, *Salvia sclarea* which is specific for Tn (Bird & Wingham, 1973). Male BALB/c mice were injected intraperitoneally at intervals of three weeks with 0.2 ml of a 5% suspension of Tn positive erythrocytes and a booster dose of antigen was given 4 days prior to hybridoma formation which was carried out using previously described techniques (Hurrell, 1982). The spleen cells from the immunized mice were fused with the mouse myeloma cell line P3.X63.Ag8 and supernatants of hybrids which had grown were screened against Tn positive and negative erythrocytes using haemagglutination and indirect fluorescence. Positive antibody producing hybrids were subcloned twice by limiting dilution and the resultant clones screened for production of anti-Tn antibodies by demonstrating reactivity against 7 examples of Tn positive erythrocytes and lack of reactivity against Tn negative erythrocytes which showed ABO, Rh, Ii, Fy, K, MNS, P, Lu, Jk, Le, Xg, Wr, En($a^-$), $M^{g+}$, $S^{D+}$, Sd($a^{++}$), St($a^+$), and T blood group specificities. From 6 anti-Tn clones one, FBT3, was selected to be further characterized and to be used in immunohistochemical studies because of its very avid reactivity with Tn positive erythrocytes. Isotyping of FBT3 by an immunodot blot technique (McDougal *et al.*, 1983) using monospecific antisera showed it to be of the IgM class. SDS-polyacrylamide-gel electrophoresis of erythrocyte membranes and immunoblotting was carried out using previously established techniques (Merry *et al.*, 1986). Haemagglutination inhibition studies were performed on culture supernatant as previously described (Springer *et al.*, 1985*b*).

### *Immunohistochemistry*

Tissue was obtained from fresh surgical specimens, embedded and frozen in OCT medium (Tissue-Tek, Miles Laboratories, USA) and stored at −80°C prior to use. The storage interval varied between 2 h and 10 months. Adjacent blocks of tissue were fixed in 4% formaldehyde in phosphate buffer (pH 7.0) and processed to paraffin blocks for routine histopathological assessment. Material was available from a variety of cases of carcinoma as shown in Table I. There were also 12 cases of non-Hodgkin's lymphoma, 1 of Hodgkin's lymphoma, and 10 reactive lymph nodes.

**Table I** Expression of Tn in normal and neoplastic tissues

| | | *Negative* | *Punctate staining* | *Cell[a] staining* | *Total* |
|---|---|---|---|---|---|
| Appendix | Normal | 1 | 1 | – | 2 |
| Anus | Carcinoma | – | – | 1 | 1 |
| Colon | Normal | 11 | 9 | – | 20 |
| | Adenoma | 2 | – | 2 | 4 |
| | Carcinoma | 1 | – | 29 | 30 |
| Ileum | Carcinoma | – | – | 1 | 1 |
| Stomach | Normal | 1 | 11 | – | 12 |
| | Leiomyoma | 1 | – | – | 1 |
| | Dysplasia | – | 1 | 4 | 5 |
| | Metaplasia | – | 1 | 1 | 2 |
| | Adenocarcinoma | 3 | – | 18 | 21 |
| Oesophagus | Normal | 2 | 1 | – | 3 |
| | Adenocarcinoma | 1 | – | 1 | 2 |
| | Squamous carcinoma | 1 | – | – | 1 |
| Gall bladder | Carcinoma | – | – | 1 | 1 |
| Liver | Normal | 1 | – | – | 1 |
| | Hepatocarcinoma | – | – | 1 | 1 |
| Breast | Normal | 3 | – | – | 3 |
| | Carcinoma | 3 | – | 21 | 24 |
| Cervix | Carcinoma | – | – | 1 | 1 |
| Endometrium | Proliferative | – | 5 | – | 5 |
| | Carcinoma | 2 | – | 1 | 3 |
| Ovary | Normal | 5 | – | – | 5 |
| | Carcinoma | 1 | – | 6 | 7 |
| Testis | Normal | 1 | – | – | 1 |
| | Teratoma | 1 | – | 1 | 2 |
| Bladder | Normal | 2 | – | – | 2 |
| | Dysplastic | – | – | 1 | 1 |
| | Carcinoma *in situ* | 1 | – | – | 1 |
| | Carcinoma T1 | – | – | 1 | 1 |
| | T2 | 5 | – | 2 | 7 |
| | T3 | – | – | 3 | 3 |
| Prostate | Benign hyperplasia | 4 | – | – | 4 |
| | Carcinoma | 4 | – | 3 | 7 |
| Lung | Normal | 5 | 1 | – | 6 |
| | Carcinoma | 8 | – | 7 | 15 |
| | Mesothelioma | 1 | – | – | 1 |
| Skin | Melanoma | 4 | – | 4 | 8 |
| | Squamous cell carcinoma | 2 | – | 1 | 3 |
| | Histiocytoma | 1 | – | – | 1 |
| Tonsil | Normal | 5 | – | – | 5 |
| | Squamous carcinoma | – | – | 1 | 1 |
| Parathyroid | Carcinoma | 1 | – | – | 1 |
| Brain | Normal | 1 | – | – | 1 |
| | Astrocytoma | 3 | – | – | 3 |

[a]Diffuse cell membrane and/or cytoplasmic staining.

The working solution of FBT3 was a 1:100 dilution of the hybridoma culture supernatant in TRIS-saline buffer, pH 7.6, and had a protein concentration of $0.05\,\mu g\,ml^{-1}$. All tissues were stained using a modification of the indirect Avidin-Biotin-Complex (ABC) technique of Hsu *et al.* (1981) which was modified by the addition of 8% $NiCl_2$ to the final diaminobenzidine (DAB)/$H_2O_2$ solution. This modification produces a final staining which is black in colour and of greater intensity than DAB alone. The sections were then counterstained in chloroform extracted methyl green which imparts a clear green colour to the nuclei. As a negative control the sections were stained with HO-2.2 an IgM antibody which is directed against the Lyt-2.2 murine T-lymphocyte differentiation antigen and which is also derived from the P3.X63.Ag8 fusion line. Sections from a specimen of gastric carcinoma known to react with FBT3 were included with each batch of unknowns as a positive control.

The results were assessed and scored by two independent observers. A positive result was recorded when dense brown/black staining was seen over defined cytological structures.

### Results

Monoclonal antibody FBT3 was shown to react specifically with Tn positive erythrocytes, by haemagglutination and indirect fluorescence testing. Minimal haemagglutination inhibition was found with N-acetyl-D-galactosamine. Specificity of this antibody was further demonstrated by immunoblotting. Figure 1 shows the immunoblot of FBT3 against normal and Tn erythrocyte membrane proteins. Binding to $\alpha$, $\delta$ and dimers of $\alpha$ and $\delta$ sialoglycoproteins in Tn positive erythrocytes was observed with FBT3, but none was evident with normal erythrocytes.

The overall immunohistochemical findings are shown in

**Figure 1** Reaction of FBT3 with membrane proteins separated by SDS/polyacrylamide-gel electrophoresis from normal and Tn erythrocytes [(a) Tn membranes, (b) membranes from normal $A_1$ erythrocytes, (c) membranes from normal group 0 erythrocytes].

**Figure 2** Normal colonic mucosa (frozen section) x 1,000. Granular Tn staining is seen in some goblet cells in a supranuclear position (arrowed). Cell membranes are not stained.

**Figure 3** Carcinoma of breast (frozen section) x 250. There is an infiltrate of poorly differentiated carcinoma. Stained for Tn (modified ABC technique) surrounding a normal breast duct (arrowed). The predominant staining here is cytoplasmic.

Table I and examples are shown in Figures 2 and 3. Fine granular supranucleur staining was seen in some normal glandular epithelia (Figure 2 and Table I). This staining corresponded to the region of the Golgi apparatus and was clearly different from that seen in cancer cells. By contrast, staining of neoplastic cells was intense and was distributed both on the cell membrane and within the cytoplasm, sometimes being greater in the perinuclear region. Frozen tissue tended to stain slightly more strongly than formalin-fixed tissue. Non-malignant mucosa immediately adjacent to gastrointestinal tumours showed occasional positive staining on the luminal border of cells but elsewhere the normal mucosa was negative apart from punctate staining as described above. In one case of gastric carcinoma the gastric carcinoma was strongly positive and a concomitant leiomyoma was negative.

Examination of normal lymphoid tissues showed faint staining of dendritic-like processes of some cells. This was seen in 6 of 10 normal reactive nodes, 3 of 12 cases of non-Hodgkin's lymphoma but not in the one case of Hodgkin's disease studied. Diffuse staining of large blast cells was seen in 1 of the 12 cases of non-Hodgkin's lymphoma.

### Discussion

The present study used a monoclonal antibody raised against the Tn epitope on erythrocytes and a sensitive immunohistochemical technique to demonstrate that an epitope the same as or similar to the Tn epitope can be expressed by malignant cells. The antigen was detected both on the surface and within the cytoplasm and, by contrast with many other tumour associated antigens, positive staining was usually quite intense and background staining insignificant. This adds to the usefulness of the antigen as a tumour marker.

The expression of T and Tn in tumours has previously been studied by Springer et al. (1979; 1983; 1985a, b). Most of their studies have involved expression of the T antigen, which has been found to be frequently expressed by cancer cells. More recently, they have shown that Tn may also be expressed by cancer cells. In the studies of Tn they principally used absorption of polyclonal human anti-Tn by tissue, decrease in antibody titre as their assay, and studied breast cancer as the prototypic cancer. They carried out limited studies using immunohistochemistry to detect binding of polyclonal human anti-Tn and reported that normal cells either did not stain or stained weakly whereas tumour cells stained much more intensely. They raised monoclonal antibodies to Tn using breast cancer tissue as the immunogen and, using histochemistry, they noted that 3 of 5 breast cancers showed positive staining, and that normal structures showed less, although often some, staining (Springer *et al.*, 1985*a*). Hirohashi *et al.* (1985) characterized as Tn two monoclonal antibodies raised against cancer cells and indicated that they labelled neoplastic tissues from cancer patients but not the corresponding normal tissues from normal individuals.

The present study used a monoclonal antibody raised against Tn positive erythrocytes and the results using this antibody showed that 104 of 147 cases of cancer were positive. We have no proof that in cancer cells the antibody was detecting the Tn epitope; it may have been detecting a closely related structure but the term Tn is used for convenience. Expression of Tn was not confined to a single type of epithelial cancer. Staining of the non-neoplastic 'normal' cells adjacent to the tumour was not seen. However, fine supra-nuclear punctate staining was seen in some normal glandular cells (Figure 2 and Table I), but the appearance was quite different from that seen in malignancy (Figure 3).

The findings in lymphomas and lymphoid tissue differed from those for epithelial tumours. Staining of dendritic processes was seen in normal and reactive lymph nodes, but diffuse cell staining was seen in only 1 case and some lymphomas.

The importance of Tn with respect to tumour biology and the relationship between tumour and host remains to be determined. It is possible that expression of Tn may be an epiphenomenon of little basic importance but several observations suggest that this may not be the case. The fact that the Tn epitope is observed on carcinoma cells arising from a variety of different tissues indicates that the epitope is not tissue specific but may have a wider importance. Cells expressing Tn on their membrane show alterations in surface charge and adhesiveness (Springer *et al.*, 1983) and such alterations could be involved in the ability of malignant cells to infiltrate and metastasize. Normal individuals can mount an immune reaction against Tn, which raises the possibility that expression of Tn may influence the interaction between tumour and host. Leukaemic cells may show expression of Tn and we have observed two patients, one with acute myeloid leukaemia in whom Tn positive cells could not be detected at initial presentation but gradually increased in number as the disease progressed until the whole leukaemic population was Tn positive at final relapse (Roxby *et al.*, 1986) and the other with myelodysplasia in whom a clone of Tn positive cells is gradually increasing in number. These findings could be interpreted as indicating a relationship between Tn expression and disease progression.

Irrespective of any more fundamental importance of the expression of Tn, the present results raise the possibility that it may provide a valuable diagnostic marker for carcinoma cells. Whether this type of application will be feasible is uncertain and will depend on the results of study of archival material and of material from pre-neoplastic tissues in addition to neoplastic and normal tissues. Both these applications are the subject of further investigation.

We thank the following individuals and institutions for supplying Tn positive erythrocytes: Dr D. Hammil; Dr W. Wagstaff; Prof. J.P. Cartron; the American Red Cross Blood Services, Huntington; the American Red Cross Blood Services, San Jose; and the Community Blood Centre, Kansis City.

This study was supported by the Flinders Medical Centre Research Foundation.

### References

BIRD, G.W.G. & WINGHAM, J. (1973). Seed agglutinin for rapid identification of Tn polyagglutination. *Lancet*, **i**, 677.

CARTRON, J.P., ANDREU, G., CARTRON, J., SALMON, C.H. & BIRD, G.W. (1978). Selective deficiency of 3-$\beta$-D-galactosyltransferase (T-transferase) in Tn-polyagglutinable erythrocytes. *Lancet*, **i**, 856.

HIROHASHI, S., CLAUSEN, H., YAMADA, T., SHIMOSATO, Y. & HAKOMORI, S.-I. (1985). Blood group A cross-reacting epitope defined by monoclonal antibodies NCC-LU-35 and -81 expressed in cancer of blood group O or B individuals: Its identification as Tn antigen. *Proc. Natl. Acad. Sci. USA*, **82**, 7039.

HSU, S.-M., RAINE, L. & FANGER, H. (1981). Use of avidin-biotin peroxidase complex in immunoperoxidase techniques. *J. Histochem. Cytochem.*, **29**, 577.

HURRELL, J.R. (1982). *Monoclonal hybridoma antibodies: Techniques and applications*. CRC Press Inc: Boca Raton, Florida.

ISSITT, P.D. (1981). *The MN blood group system*. Montgomery Scientific Publications: Ohio.

McDOUGAL, J.S., BROWNING, S.W., KENNEDY, S. & MOORE, D.D. (1983). Immunodot assay for determining isotype and light chain type of murine monoclonal antibodies in unconcentrated hybridoma culture supernates. *J. Immunol. Methods*, **63**, 281.

MERRY, A.H., HODSON, C., THOMSON, E., MALLINSON, G. & ANSTEE, D.J. (1986). The use of monoclonal antibodies to quantify the levels of sialoglycoproteins $\alpha$ and $\delta$ and variant sialoglycoproteins in human erythrocyte membranes. *Biochem. J.*, **233**, 93.

ROXBY, D.J., MORLEY, A.A. & BURPEE, M. (1987). Detection of the Tn antigen in leukaemia using monoclonal anti-Tn antibody and immunohistochemistry. *Br. J. Haem.* (in press).

SPRINGER, G.F. (1984). T and Tn, general carcinoma antigens. *Science*, **224**, 1198.

SPRINGER, G.F., CHEINGSONG-POPOV, R., SCHIRRMACHER, V., DESAI, P.R. & TEGTMEYER, H. (1983). Proposed molecular basis of murine tumor cell-hepatocyte interaction. *J. Biol. Chem.*, **258**, 5702.

SPRINGER, G.F., DESAI, P.R., MURTHY, M.S., TEGTMEYER, H. & SCANLON, E.F. (1979). Human carcinoma-associated precursor antigens of the blood group MN system and the hosts immune response to them. *Prog. Allergy*, **26**, 42.

SPRINGER, G.F., TAYLOR, C.R., HOWARD, D.R. & 5 others (1985*a*). Tn, a carcinoma-associated antigen reacts with anti-Tn of normal human sera. *Cancer*, **55**, 561.

SPRINGER, G.F. & DESAI, P.R. (1985*b*). Tn epitopes, immunoreactive with ordinary anti-Tn antibodies on normal, desialylated human erythrocytes and on Thomsen–Friedenreich antigen isolated therefrom. *Mol. Immunol.*, **22**, 1303.